# A Novel *Microviridae* Phage (CLasMV1) From “*Candidatus* Liberibacter asiaticus”

**DOI:** 10.3389/fmicb.2021.754245

**Published:** 2021-10-13

**Authors:** Ling Zhang, Ziyi Li, Minli Bao, Tao Li, Fang Fang, Yongqin Zheng, Yaoxin Liu, Meirong Xu, Jianchi Chen, Xiaoling Deng, Zheng Zheng

**Affiliations:** ^1^Guangdong Province Key Laboratory of Microbial Signals and Disease Control, South China Agricultural University, Guangzhou, China; ^2^China-United States Citrus Huanglongbing Joint Laboratory, National Navel Orange Engineering Research Center, Gannan Normal University, Ganzhou, China; ^3^Horticulture Research Institute, Guangxi Academy of Agricultural Sciences, Nanning, China; ^4^United States Department of Agriculture, San Joaquin Valley Agricultural Sciences Center, Agricultural Research Service, Parlier, CA, United States

**Keywords:** “*Candidatus* Liberibacter asiaticus,”, *Microviridae*, phage, genomic characterization, prevalence, evolution

## Abstract

“*Candidatus* Liberibacter asiaticus” (CLas) is an unculturable phloem-limited α-proteobacterium associated with citrus Huanglongbing (HLB; yellow shoot disease). HLB is currently threatening citrus production worldwide. Understanding the CLas biology is critical for HLB management. In this study, a novel single-stranded DNA (ssDNA) phage, CLasMV1, was identified in a CLas strain GDHZ11 from Guangdong Province of China through a metagenomic analysis. The CLasMV1 phage had a circular genome of 8,869 bp with eight open reading frames (ORFs). While six ORFs remain uncharacterized, ORF6 encoded a replication initiation protein (RIP), and ORF8 encoded a major capsid protein (MCP). Based on BLASTp search against GenBank database, amino acid sequences of both MCP and RIP shared similarities (coverage > 50% and identity > 25%) to those of phages in *Microviridae*, an ssDNA phage family. Phylogenetic analysis revealed that CLasMV1 MCP and RIP sequences were clustered with genes from CLas and “*Ca*. L. solanacearum” (CLso) genomes and formed a unique phylogenetic lineage, designated as a new subfamily *Libervirinae*, distinct to other members in *Microviridae* family. No complete integration form but partial sequence (∼1.9 kb) of CLasMV1 was found in the chromosome of strain GDHZ11. Read-mapping analyses on additional 15 HiSeq data sets of CLas strains showed that eight strains harbored complete CLasMV1 sequence with variations in single-nucleotide polymorphisms (SNPs) and small sequence insertions/deletions (In/Dels). PCR tests using CLasMV1-specific primer sets detected CLasMV1 in 577 out of 1,006 CLas strains (57%) from southern China. This is the first report of *Microviridae* phage associated with CLas, which expands our understanding of phage diversity in CLas and facilitates current research in HLB.

## Introduction

“*Candidatus* Liberibacter asiaticus” (CLas) is an unculturable phloem-limited α-proteobacterium associated with citrus Huanglongbing (HLB; also known as yellow shoot disease), a highly destructive disease in citrus production worldwide. Knowledge about CLas biology was limited due to the inability to culture it *in vitro*. However, recent development in high-throughput sequencing technology has opened a new venue for research in CLas (Duan et al., 2009; Zheng et al., 2014b). Among the new discoveries are CLas phages, i.e., viruses infecting CLas (Zhang et al., 2011; Zheng et al., 2018; Dominguez-Mirazo et al., 2019). Analyses of these phage genomes have significantly enriched the biological information of CLas.

Typically, a phage particle is composed of a protein capsid and a circular DNA genome (Adams, 1959). Since CLas titers are generally low, observations on phages particles using electron microscopy are challenging (Zhang et al., 2011). However, with the aid of high-throughput sequencing technology, the circular phage DNA genome could be detected (Zhang et al., 2011; Zheng et al., 2018). Based on the presence of circular plasmid form genomic DNA, three types of phages/prophages (Type 1, Type 2, and Type 3) have been described (Zhang et al., 2011; Zheng et al., 2018). Type 1 prophage (represented by SC1) was reported to be involved in the lytic cycle of forming phage particles; Type 2 prophage (represented by SC2) was found to be involved in the lysogenic conversion of CLas pathogenesis (Fleites et al., 2014; Jain et al., 2015); and Type 3 prophage (represented by prophage P-JXGC-3) carried a restriction-modification system (R-M system) (Zheng et al., 2018). Variants within a phage type have been reported in CLas strains from Pakistan (Cui et al., 2020). It is in general believed that more CLas phages remain to be discovered.

Most characterized phages possessed double-stranded DNA (dsDNA) genome as three known phage types in CLas (Zhang et al., 2011; Zheng et al., 2018). However, recent metagenomic studies demonstrate that phages with single-stranded DNA (ssDNA) genomes, especially for those in the family of *Microviridae*, are more common in many bacteria (Desnues et al., 2008; Tucker et al., 2010; Labonté and Suttle, 2013; Székely and Breitbart, 2016). *Microviridae* phages had a 4.4∼6.1 kb circular ssDNA genome enclosed within small icosahedral capsid (∼30 nm) (Székely and Breitbart, 2016). The phage replicates using a rolling-circle mechanism associated with a circular dsDNA, named as replicative form or RF that can be detected by standard DNA research techniques.

All *Microviridae* phages encoded two relatively conserved proteins, a capsid protein and a replication initiator protein (Cherwa and Fane, 2011). Currently, *Microviridae* is mainly classified into two subfamilies, *Bullavirinae* and *Gokushovirinae*, and an unclassified group (Cherwa and Fane, 2011; Krupovic et al., 2016). *Bullavirinae* contain 11 genes with genome size from 5.4 to 6 kb, typified by the bacteriophage phiX174 (Sanger et al., 1977). *Gokushovirinae* have genome ranging from 4.4 to 4.9 kb including nine genes and are specialized to infect intracellular parasites such as phage SpV4 infecting *Spiroplasma citri*, the pathogen of citrus stubborn disease (Saglio et al., 1973).

Here, we reported a novel *Microviridae* phage, CLasMV1, discovered through a metagenomic analysis of CLas strain GDHZ11 from China. CLasMV1 had a circular genome with eight open reading frames (ORFs): one encoded a unique major capsid protein (MCP), one encoded a replication initiation protein (RIP), and the encoding status of the other ORFs remained unknown. Phylogenetic analysis revealed that CLasMV1 represent a newly recognized lineage, designated as subfamily *Libervirinae*, along with *Bullavirinae* and *Gokushovirinae* under *Microviridae* family. A partial CLasMV1 sequence was found in CLas strain GDHZ11 chromosome. Population diversity and biological significance of CLasMV1 in CLas were further analyzed and discussed.

## Materials and Methods

### Source of *Candidatus* Liberibacter asiaticus Strains

Citrus leaves with typical HLB symptoms from seven citrus cultivars were collected from nine provinces in China between March 2016 and November 2019 (Table 1). DNA was extracted from each sample following protocols published previously (Zheng et al., 2014b). Briefly, plant total DNA was extracted by using E. Z. N. A. HP Plant DNA Kit (OMEGA Bio-Tek Co., Guangdong, China). The presence of CLas in DNA sample was confirmed by TaqMan Real-time PCR with primer-probe set (RNRf/RNRp/RNRr) (Zheng et al., 2016) or CLas4G/HLBp/HLBr (Bao et al., 2020).

**TABLE 1:** A summary of “*Candidatus* Liberibacter asiaticus” (CLas) sample collection in China and CLasMV1 related information.

No.	Geographical origin	No. of samples			Copy number of CLasMV1 per CLas cell (No. of samples)*				
			
		CLas	CLasMV1	Frequency	<1	1	>1	Range	Average
1	Guangdong	248	169	68%	8	6	155	0.35∼285	15.6
2	Guangxi	272	203	75%	18	15	170	0.54∼1,178	67.5
3	Jiangxi	69	51	74%	2	4	45	0.68∼206	26.4
4	Hunan	81	63	78%	1	4	58	0.82∼420	82.3
5	Fujian	45	38	84%	1	1	36	0.86∼37	6.1
6	Yunnan	125	19	15%	7	1	11	0.75∼40	4.5
7	Hainan	49	8	16%	1	1	6	0.28∼19	4.4
8	Guizhou	60	19	32%	0	0	19	4∼867	245.1
9	Zhejiang	57	7	12%	0	0	7	3∼12	4.7
	Total	1,006	577	57%	38	32	507	0.35∼1,178	50.7

*Copy number (R) of CLasMV1 per CLas cell is calculated based on the ΔCt method, i.e., R = 2^–ΔCt^, ΔCt = Ct (CLasMV1-1F/CLasMV1-1R) - Ct (CLas-4G/HLBr) + 1.585.

### Illumina Sequencing and Assembly

CLas strain GDHZ11, originally collected from an HLB-affected citrus tree (*Citrus reticulata* Blanco cv. Shatangju) located in Huizhou City of Guangdong Province, was enriched by the dodder-mediated CLas enrichment system developed previously (Li et al., 2021). Total DNA of CLas-enriched dodder was extracted and used for genome sequencing. Sequencing data of strain GDHZ11 were initially used for analyses of CLas-associated novel sequences. For further analyses, 11 additional CLas DNA samples from Guangdong and Yunnan provinces were selected and used for whole-genome sequencing (Table 2).

**TABLE 2:** General information of CLasMV1 phage strains from China.

No.	Strain name	Source plant	Geographical origin (city, province)	Tissue type	Total size (bp)	Accession number	GC%
1	GDHZ11	*Citrus reticulata* Blanco cv. Shatangju	Huizhou, Guangdong	Dodder	8,869	CP045566.1	36.8
2	A4	*C. reticulata* Blanco cv. Shatangju	Guangzhou, Guangdong	Midribs	8,869	MN890136	36.8
3	GDCZ2	*C. reticulata* Blanco cv. Tankan	Chaozhou, Guangdong	Fruit pith	8,696	MN890137	36.9
4	GDDQ6	*C. reticulata* Blanco cv. Gongkan	Deqing, Guangdong	Fruit pith	8,869	MN890138	36.8
5	GDDQ7	*C. reticulata* Blanco cv. Gongkan	Deqing, Guangdong	Fruit pith	8,690	MN890139	36.9
6	GDQY1	*C. reticulata* Blanco cv. Shatangju	Qingyuan, Guangdong	Midribs	8,696	MN890140	36.9
7	GDXH1	*C. reticulata* Blanco cv. Chachiensis	Xinhui, Guangdong	Fruit pith	8,851	MN890141	36.8
8	YNJS5	*C. reticulata* Blanco cv. Maogukan	Jianshui, Yunnan	Fruit pith	8,885	MN890142	36.7
9	YNJS47	*C. sinensis*	Jianshui, Yunnan	Midribs	8,869	MN890143	36.8

All genome sequencing was performed with Illumina HiSeq platform (Illumina Inc., San Diego, CA, United States) after PCR-free library construction. For dodder-enriched CLas samples, the HiSeq data were filtered with whole-genome sequence of *Cuscuta australis* (NQVE00000000.1) and *Cuscuta campestris* (OOIL00000000.1) using Bowtie2 software (Langmead and Salzberg, 2012). For citrus-CLas samples, the HiSeq data were filtered with the genome sequences of *Citrus sinensis* (AJPS00000000.1), *Citrus clementina* (AMZM00000000.1), *C. sinensis* mitochondrion (NC_037463.1), and *C. sinensis* chloroplast genome (DQ864733.1) by using Bowtie2 software. All mapped reads were removed, and only the unmapped reads were retained for further assembly.

*De novo* assembly was performed by CLC Genomic Workbench v9.5 (QIAGEN Bioinformatics, Aarhus, Denmark) with default setting. The ordering of CLas contigs was performed by using all *de novo* assembly contigs to BLAST against CLas strain A4 genome (CP010804.2) through standalone BLAST software (word_size = 28, *e*-value = 1e-5) (Camacho et al., 2009). For prophage region, the reference-guided assembly was performed by using three phage sequences (SC1, SC2, and P-JXGC-3) as reference. The draft CLas genome sequence was generated from the combination of *de novo* assembly and reference-guide assembly.

### Identification and Validation of the *Candidatus* Liberibacter asiaticus-Associated Novel Sequence

To identify the novel CLas-associated sequence, all *de novo* assembly contigs from GDHZ11 HiSeq data were used to BLASTn (word_size = 28, *e*-value = 1e-5) against 25 available CLas genomes and three phage sequences (SC1, SC2, and P-JXGC-3) (Supplementary Table 1). Contigs with partial match (>500 bp of non-matched sequence) were retrieved from the blast result and used for contig extension. The reads (150 bp for each) from two ends of candidate novel CLas contig were collected and used as query to BLASTn against *de novo* contigs of GDHZ11 by using Standalone BLAST software (word size = 16, *e*-value = 10) (Camacho et al., 2009). Contigs with > 95% identity were selected and used for contig extension. A circular sequence would be found through contig overlapping extensions that connected the 3′ end of a contig to the 5′ end of the same contig. The overlapped regions were further confirmed with standard PCR-Sanger’s sequencing method. Primers were designed with Primer 3 server (Untergasser et al., 2012; Supplementary Table 2). The expected PCR amplicon were sequenced by Sanger’s sequencing after cloning with *pEASY*-*T1* plasmid (TransGen Biotech, Beijing, China). Amplicon sequences were assembled using SeqMan software.^1^

Sequence circularity was further evaluated by an *in silico* “Reads Mapping Test” approach described previously (Zheng et al., 2018). Briefly, the first 4,000 bp at the 5′ end of circular sequence was cut and attached to the 3′ end to generate a new circular sequence. For comparison, a 4,000-bp sequence randomly selected from the chromosomal region of CLas GDHZ11 genome was cut and added to the end of the circular sequence (without the first 4,000 bp) that generated another sequence to serve as the reference. Both two sequences were used as reference for read-mapping with GDHZ11 HiSeq reads. A continuous read coverage (no gap) of the connection region suggested sequence circularity (Supplementary Figure 1).

### Gene Annotation and Phylogenetic Analysis

Annotation of sequence was performed on RAST server (Aziz et al., 2008). All predicted ORFs were further analyzed through BLASTx against the non-redundant protein sequences in National Center for Biotechnology Information (NCBI) GenBank database (version 235.0) and the NCBI conserved domain database (CDD, v3.17). The homologous genes between phage and CLas were identified by using each phage ORFs as query to BLASTn against CLas genome sequences (Supplementary Table 1). All predicted protein sequences were further used to BLAST against the Viruses database (taxid:10239) and the Plasmids database (taxid:36549) in NCBI GenBank database (version 235.0) through NCBI web Quick BLASTp with default setting (expect threshold = 10 and word size = 6).

MCP and RIP sequences from representative ssDNA phages under *Microviridae* family and top BLAST hits toward the MCP and RIP genes of candidate phage were selected for phylogenetic analyses (Supplementary Table 1). The complete amino acid sequences of MCP and RIP were aligned by MEGA6 using default setting (Tamura et al., 2013). Phylogenetic trees were constructed using MEGA6 with maximum-likelihood method and bootstrap value of 500 (Tamura et al., 2013).

### Identification and Comparison of CLasMV1 From Different *Candidatus* Liberibacter asiaticus Strains

As described later, CLasMV1 was the phage identified in CLas strain GDHZ11 in this study. In addition to 12 CLas HiSeq data sequenced in this study, four additional HiSeq/MiSeq data (A4, HHCA, FL17, and SGCA5) from our previous studies (Zheng et al., 2014a, b, 2015, 2017) were also selected for identification of phage through read-mapping approach (Table 2). Briefly, the HiSeq data were directly mapped to candidate phage sequence through CLC Genomic Workbench v9.5 (QIAGEN Bioinformatics, Aarhus, Denmark) with default setting. The presence of candidate phage was directly extracted from the mapping result when the fraction of reference covered over 80. Whole-genome comparison of phages from different CLas samples was performed with standalone BLAST software (word_size = 28, *e*-value = 1e-5) (Camacho et al., 2009). Sequence variants, including SNPs and In/Dels, were directly identified from BLAST result.

For CLas DNA samples collected from nine provinces in southern China, the presence of CLasMV1 phage was identified by CLasMV1-specific real-time PCR as described below. In addition to CLasMV1, the first *Microviridae* phage was identified in CLas strain GDHZ11, and others identified by phage were named by adding the sample name after an underscore following the phage name. For example, CLasMV1_GDDQ6 denoted phage CLasMV1 strain GDDQ6 (from CLas sample GDDQ6).

### Development of CLasMV1-Specific Real-Time PCR

All phage sequences obtained in this study were aligned with MEGA6 to identify the phage conserved region (Tamura et al., 2013). The phage-specific primer sets (CLasMV1-1F/CLasMV1-1R and CLasMV1-2F/CLasMV1-2R) were designed based on the unique and conserved region of phage sequence using Primer 3 server (Untergasser et al., 2012; Supplementary Table 2). The specificity of primers was evaluated by using the primer sequence to blast against with NCBI nucleotide database through NCBI web BLASTn (expect threshold = 10 and word size = 16). In addition, the specificity of primers was also verified with real-time PCR by using a GDHZ11 DNA sample (CLasMV1-positive) and CLas-free DNA samples extracted from healthy citrus samples and dodder tendrils.

The SYBR Green Real-time PCR assays were performed in CFX Connect Real-Time System (Bio-Rad, Hercules, CA, United States). The reaction mixture contained 1 μl of DNA template (∼25 ng), 0.5 μl of each forward and reverse primer (10 μM), and 10 μl of iQ^TM^ SYBR^®^ Green Supermix (Bio-Rad) in a final volume of 20 μl with the following procedure: 95°C for 3 min, followed by 40 cycles at 95°C for 10 s and 60°C for 30 s. Fluorescence signal was captured at the end of each 60°C step, followed by a melting point analysis.

### Evaluation of CLasMV1 Copy Number

For CLas samples sequenced with Illumina platform, the copy number of CLasMV1 was evaluated by comparison of the average nucleotide coverage (ANC) of CLasMV1 sequence and CLas chromosome through read-mapping. Briefly, the HiSeq data were directly mapped to CLas strain A4 chromosomal region (CP010804.2, from nucleotide position of 1 to 1,191,644) and CLasMV1 sequence (CP045566.1) by CLC Genomic Workbench v9.5 (QIAGEN Bioinformatics, Aarhus, Denmark) with default setting. The ANC of CLas chromosomal region and CLasMV1 phage sequence was calculated with the following equation: ANC = total bases of all aligned reads/the length of the consensus sequence. The copy number of CLasMV1 was indicated as the copy number of CLasMV1 per CLas cell, i.e., the copy number of CLasMV1 = the ANC of CLasMV1 sequence/the ANC of CLas chromosomal region.

For other CLas samples not sequenced, the copy number of CLasMV1 was evaluated by relative quantitative real-time PCR with the CLasMV1-specific primer set and CLas-specific primer set. The density of CLasMV1 was indicated as the copy number of CLasMV1 per CLas cell with the ΔCt method (Wittwer et al., 2001), i.e., R = 2^–ΔCt^, ΔCt = Ct (CLasMV1-1F/CLasMV1-1R) - Ct (primer set targeted single copy gene). The Ct value generated by primer set targeted single copy gene was converted from the Ct value generated by primer set CLas4G/HLBr targeted three copies of 16S rRNA gene with the following equation: Ct (primer set targeted single copy gene) = Ct (CLas4G/HLBr) + X, where X is 1.585.

## Results

### Detection of CLasMV1

**FIGURE 1:**
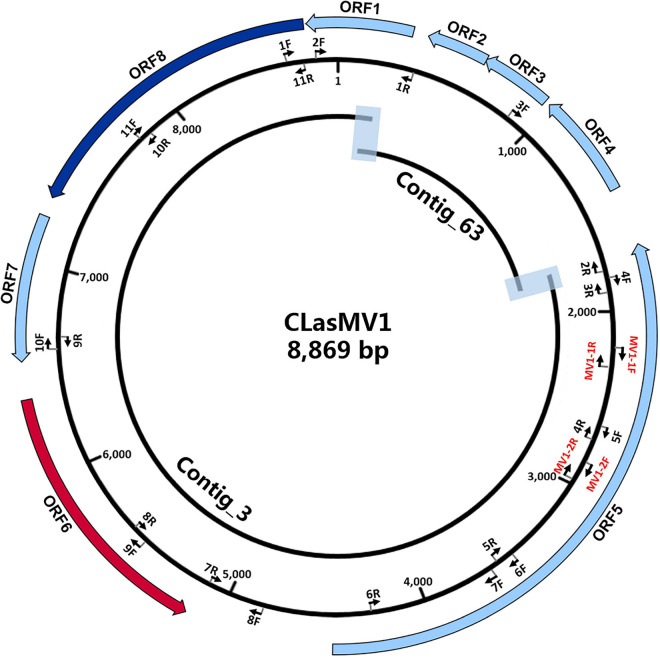
Schematic representation of genome assembly of CLasMV1 phage. Outside ring: open reading frame (ORF) annotations. ORF6 (marked in red arrow) = replication initiation protein (RIP) gene; ORF8 (marked in blue arrow) = major capsid protein (MCP) gene. Middle ring: arrow pairs (nF–nR) are primer sets for gap closure and Sanger sequencing confirmations; arrow pairs in red (MV1-1F–MV1-1R and MV1-2F–MV1-2R) are CLasMV1 specific primer sets. All primers sequences are listed in Supplementary Table 2. Inside ring: Contig_3 and Contig_63 with overlapped regions highlighted.

The HiSeq platform yielded a total of 67,133,182 short reads (150 bp) derived from GDHZ11 DNA sample. A total of 900 contigs (> 1 kb) were generated from *de novo* assembly. Twenty-eight contigs had 100% length coverage and > 98% identity to the referenced CLas genome sequences (Supplementary Table 3). One contig (Contig_3 with 7,324 bp) showed 59% length coverage and 99% identity (Supplementary Table 3). Contig extension of Contig_3 sequence showed that the 3′ end of Contig_3 was connected to 5′ end of Contig_63 (1,379 bp), while the 3′ end of Contig_63 was connected back to the 5′ end of Contig_3, forming an 8,869-bp circular DNA molecule (Figure 1). Read-mapping test further confirmed the circularity of Contig_3–Contig_63 sequence (Supplementary Figure 1). In addition, the full length of Contig_3–Contig_63 sequence was also confirmed by PCR and Sanger sequencing (Supplementary Figure 2 and Supplementary Table 2).

**FIGURE 2:**
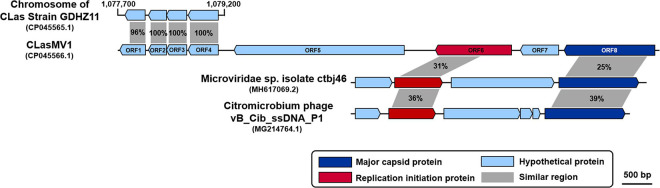
Comparison of amino acid sequences among CLasMV1, homologs in “*Candidatus* Liberibacter asiaticus” (CLas) strain GDHZ11 chromosome, and representative *Microviridae* phages. Percentage similarities are marked within gray shadows.

RAST annotation of the circular Contig_3–Contig_63 sequence detected eight ORFs (Figure 1 and Table 3). BLASTp search using amino acid sequences of each ORF against NCBI Plasmid (taxid:36549) and Viruses database (taxid:10239) showed that ORF6 encoded a RIP and ORF8 encoded an MCP in *Microviridae*, an ssDNA phage family (Figure 2 and Table 3). The genetic nature of the other six ORFs remained as putative proteins. Based on HiSeq read-mapping, the ANC of Contig_3 was 305 × and that of Contig_63 was 342 × (Supplementary Table 3). These were in contrast to the ANC of ∼65 × of the CLas chromosome (Supplementary Table 3), leading to about five copies of the circular DNA per CLas cell calculated.

**TABLE 3:** General information of open reading frames (ORFs) in CLasMV1 genome.

Name	Locus_tag	Start	End	Nucleotide (bp)	Amino acid (aa)	BLASTp with NCBI conserved domain database		BLAST result (matched length in amino acid/similarity %)*		Annotation
								
						Conserved domain	Domain ID	CLas genome	Viruses + plasmids	
ORF1	GE519_gp01	351	8,735	486	161	NF	NF	WP_144299319 (161/100%)	NF	Hypothetical protein
ORF2	GE519_gp02	701	420	282	93	NF	NF	WP_015452912.1 (93/100%)	NF	Hypothetical protein
ORF3	GE519_gp03	1,024	698	327	108	NF	NF	WP_015452913.1 (102/100%)	NF	Hypothetical protein
ORF4	GE519_gp04	1,530	1,048	483	160	NF	NF	WP_015452914.1 (160/100%)	NF	Hypothetical protein
ORF5	GE519_gp05	4,451	1,797	2,655	884	NF	NF	WP_015452962.1 (109/99%)	NF	Hypothetical protein
ORF6	GE519_gp06	6,365	5,148	1,218	405	Replication initiation protein	PHA00330	WP_015452965.1 (405/100%)	ATW62975.1 (71/37%)	Replication initiation protein
ORF7	GE519_gp07	6,534	7,208	675	224	2OG-FeII_Oxy	cl21496	WP_015452962.1 (44/86%)	NF	Hypothetical protein
ORF8	GE519_gp08	8,725	7,298	1,428	475	NF	NF	WP_045490387.1 (447/99%)	QJB21054.1 (112/25%)	Major capsid protein

NF, not found; NCBI, National Center for Biotechnology Information. *Only the best match was listed here.

Considering the similarity of ORF6 (37%) and ORF8 (25%) to members of *Microviridae* (Table 3), the circular Contig_3–Contig_63 sequence could be taken as the RF of a not-yet reported ssDNA phage, designated as CLasMV1. CLasMV1 phage genome had a G + C content of 36.8%. The CLasMV1 sequence had been deposited in GenBank under the accession number of CP045566.1.

### Phylogenetic Analysis of CLasMV1

**FIGURE 3:**
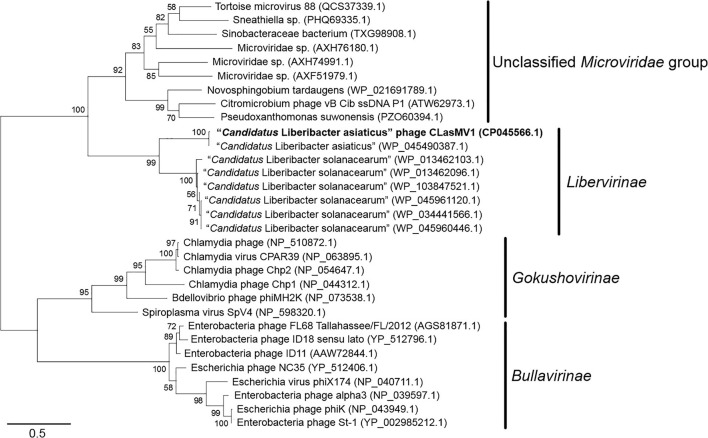
Unrooted maximum-likelihood phylogenetic analyses of CLasMV1 phage based on the full-length major capsid protein (MCP) sequence. MCP sequences from representative members of *Microviridae* and top BLAST hits toward the replication initiation protein (RIP) of CLasMV1 phage are selected for phylogenetic analyses. Bootstrap value = 500. Note the proposed subfamily *Libervirinae*.

**FIGURE 4:**
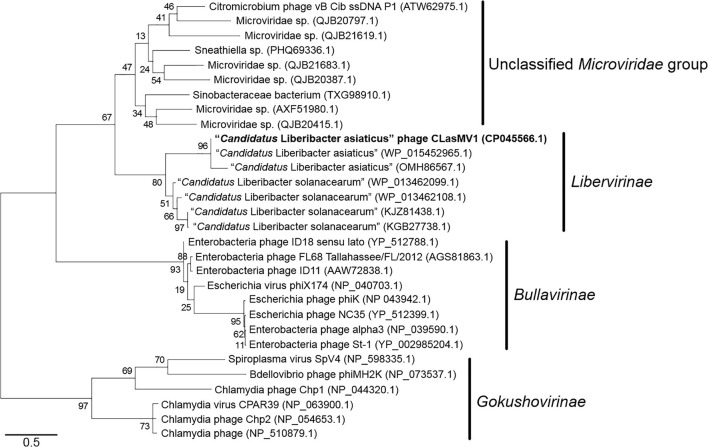
Unrooted maximum-likelihood phylogenetic analyses of CLasMV1 phage based on the full-length replication initiation protein (RIP) sequence. RIP sequences from representative members of *Microviridae* and top BLAST hits toward the RIP of CLasMV1 phage are selected for phylogenetic analyses. Bootstrap value = 500. Note the proposed subfamily *Libervirinae*.

Phylogenetic analyses of MCP and RIP sequences showed that CLasMV1 was distantly related to the known ssDNA phages from two subfamilies (*Gokushovirinae* and *Bullavirinae*) of *Microviridae* (Figures 3, 4). Both two sequences were clustered with homologous genes from CLas genome. Homologs of CLasMV1 MCP and RIP were also found in multiple genome sequences of “*Ca*. L. solanacearum” (CLso), a bacterium associated with potato zebra chip disease, but constituted a different phylogenetic cluster (Figures 3, 4). The CLas cluster and CLso cluster formed a unique phylogenetic lineage, designated as a new subfamily *Libervirinae*, distinct to other members in *Microviridae* family (Figures 3, 4).

### Genomic Diversity of CLasMV1 Strains

**FIGURE 5:**
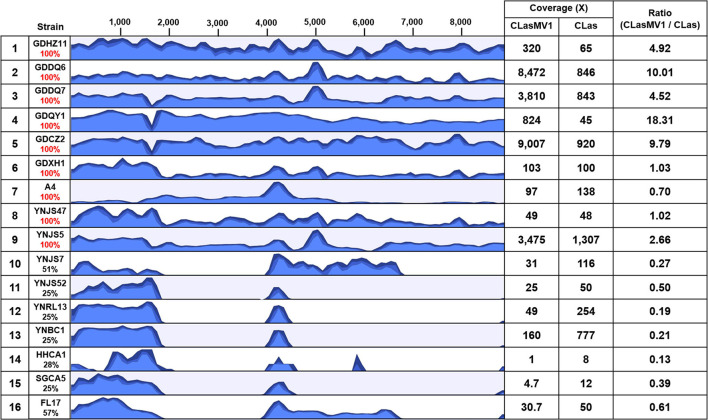
Results of read-mapping to CLasMV1 sequence (8,869 bp) with HiSeq data from 16 “*Candidatus* Liberibacter asiaticus” (CLas) strains. Row 1, reference CLas strain GDHZ11; Rows 2–13, CLas strains from China (2–7 from Guangdong Province and 8–13 from Yunnan Province). Row 14–16, CLas strains from the United States (14–15 from California and 16 from Florida). Percentages of CLasMV1 length covered are listed in column 2. The HiSeq data of CLas strains detected full length (100%) of CLasMV1 are highlighted in red in column 2. Column 3, read-mapping track. Blue graph represents read coverage in log scale. Column 4, average nucleotide coverages (ANCs) to CLasMV1 and CLas chromosome; and Column 5, ratio of ANCs inferring copy numbers of CLasMV1 in each CLas strain.

**FIGURE 6:**
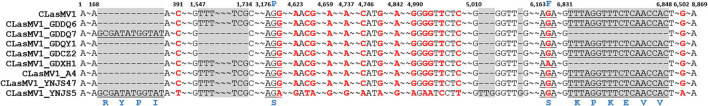
A list of single-nucleotide polymorphisms (SNPs) and small insertions/deletions (In/Dels) in nine CLaMV1 strains from southern China. The numbers above the alignment graph are sequence position reference to CLasMV1 (CP045566.1). The SNPs are highlighted in red. The In/Dels are highlighted by gray shadow. The corresponding codon located in SNPs and In/Dels are underlined with amino acids in blue. “–” represents the nucleotide deletion. “∼” represents omitted identical nucleotides.

With the HiSeq reads data of 15 CLas strains from China and the United States, eight showed the 100% coverage of CLasMV1 sequence, including six from Guangdong Province and two from Yunnan Province, but none from the United States (Figure 5). The genome size of the eight CLasMV1 strains ranged from 8,690 to 8,885 bp (Table 2). Sequence comparison of the CLasMV1 strains identified a total of 22 sequence variants, including 17 single-nucleotide polymorphisms (SNPs) and five Insertion/Deletions (In/Dels) (Figure 6). Two SNPs (nucleotide positions of 3,176 and 6,163) and two In/Dels (nucleotide positions of 168 and 6,831∼6,848) were located in the gene coding region (ORF1, 5, 6, and 7) reference to CLasMV1 genome (CP045566.1), and none of them caused the frame shifts (Figure 6). The 12-bp insertion at nucleotide position 168 of CLasMV1 genome was detected in the CLasMV1_GDDQ7 and CLasMV1_YNJS5 sequences and increased the corresponding protein by four amino acids in comparison with ORF1 of CLasMV1 (Figure 6). Conversely, an 18-bp deletion between nucleotide positions 6,831 and 6,848 of CLasMV1 was identified in CLasMV1_GDXH1 and CLasMV1_GDDQ7 sequence by reducing six amino acids of the corresponding protein as compared with the ORF7 of CLasMV1 (Figure 6). In addition, a 186-bp deletion between nucleotide positions 1,547 and 1,734, a non-coding region in CLasMV1, was identified in the CLasMV1_GDQY1, GDCZ2, and GDDQ7 sequences.

The copy number of CLasMV1, as estimated by the ratio of ANC between CLasMV1 and CLas, varied among nine CLas strains (Figure 5). The multiple copies of CLasMV1 per CLas cell were observed in six CLas strains (strains GDHZ11, GDDQ6, GDDQ7, GDQY1, GDCZ2, and YNJS5) (Figure 5). A nearly single copy of CLasMV1 per CLas cell was detected in CLas strains GDXH1 and YNJS47 with the CLasMV1/CLas ratio of 1.02 and 1.03, respectively. In addition, a CLasMV1/CLas ratio of 0.70 was observed in CLas A4 strain.

### Partial CLasMV1 Sequences in *Candidatus* Liberibacter asiaticus Chromosome

**FIGURE 7:**
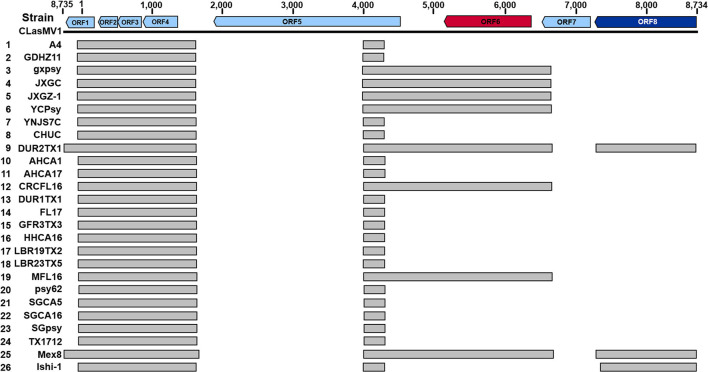
BLASTn detection of CLasMV1 sequence in “*Candidatus* Liberibacter asiaticus” genomes available in GenBank database. The homologous sequences of CLasMV1 detected in CLas strain are marked by the gray box. Rows 1–8, from China; Rows 9–24, from the United States; Row 25, from Mexico; and Row 26, from Japan. GenBank accession numbers in the order are as follows: 1, CP010804.2; 2, CP045565.1; 3, CP004005.1; 4, CP019958.1; 5, VIQL00000000.1; 6, LIIM00000000.1; 7, QXDO00000000.1; 8, VTLV00000000.1; 9, VTLS00000000.1; 10, CP029348.1; 11, VNFL00000000.1; 12, VTLW00000000.1; 13, VTLT00000000.1; 14, JWHA00000000.1; 15, VTLR00000000.1; 16, VTLY00000000.1; 17, VTMA00000000.1; 18, VTMB00000000.1; 19, VTLX00000000.1; 20, CP001677.5; 21, LMTO00000000.1; 22, VTLZ00000000.1; 23, QFZJ00000000.1; 24, QEWL00000000.1; 25, VTLU00000000.1; and 26, AP014595.1. ORF6 in red encoding major capsid protein (MCP) and ORF8 in deep blue encoding replication initiation protein (RIP).

As shown in Figure 5, seven out of the 15 CLas strains (four from Yunnan Province, China, and three from the United States) did not harbor a complete CLasMV1 sequence. Instead, sections of CLasMV1 were detected in CLas genomes. Interestingly, BLASTn search with CLasMV1 as a query against the 25 representative whole-genome sequences of CLas deposit in GenBank database also showed partial sequence hits (Figure 7). The hit regions (represented by CLasMV1 positions) were summarized into three main sections: A, positions 1–1,752, (ORF1–4); B, positions 4,081–6,683 (ORF6, partial ORF5, and ORF7); and C, positions 7,332–8,734 (ORF8). Region A was shared by all CLas strains, while Regions B and C were only found in some CLas strains completely or partially (Figure 7). In addition to the blast result, the read-mapping test also confirmed the presence of homologous regions between CLas strain GDHZ11 chromosome and CLasMV1 genome (Supplementary Figure 3).

### Frequency and Copy Number of CLasMV1 in *Candidatus* Liberibacter asiaticus Population in China

To further investigate the prevalence of CLasMV1, a total of 1,006 CLas strains samples were collected from southern China. The presence of CLasMV1 was detected by real-time PCR assay with CLasMV1-specific primer sets (Supplementary Table 2). PCR result showed that CLasMV1 was detected in 577 out of 1,006 CLas strains (577/1006, 57%) (Table 1). CLas population from nine provinces in China can be divided into two groups, the high-frequency group (>50%), including Guangdong (68%), Guangxi (75%), Jiangxi (74%), Hunan (78%), and Fujian (84%) provinces, and low-frequency group (<50%), including Yunnan (15%), Hainan (16%), Guizhou (32%), and Zhejiang (12%). The density of CLasMV1 varied among CLas strains, ranging from 0.35 to 1,178 copies per CLas cell with an average of 50.7 copies per CLas cell (Table 1). Of 577 CLas strains that harbored CLasMV1, 507 (507/577, 88%) contained multiple copies of CLasMV1 per CLas cell, and 32 CLas samples (32/577, 6%) harbored nearly a single copy of CLasMV1 per CLas cell (Table 1). Conversely, less than one copy of CLasMV1 per CLas cell was only observed in 38 CLas samples.

## Discussion

The circular CLasMV1 phage genome was generated from an assembly of two contigs (Contig_3 and Contig_63) rather than directly from a single contig. Contig_63 (1,379 bp) was a repetitive sequence that presents in both CLasMV1 phage and CLas genome (Figure 2). The assembly of long repetitive sequence using only Illumina short reads (150 bp for each read in this study) remains challenging due to assembly collapse (Tørresen et al., 2019), which can explain the disconnection of Contig_3 and Contig_63 during *de novo* assembly process. In this study, Contig_63 was first identified as a phage sequence candidate through BLAST analysis. The “contig extension” on Contig_63 was critical to resolve the *de novo* assembly collapse. However, as sequencing technology advances, long-read sequencing technologies, such as Pacific Biosciences or Oxford Nanopore Technologies, can be employed to obtain a single contig phage sequence.

Evidence for the identification of CLasMV1 as a member in *Microviridae* family was mainly based on similarities of MCP and RIP to available GenBank database (Figure 2). Especially, the CLasMV1 RIP contained a conserved domain PHA0030 (Table 3), which only occurred in members of *Microviridae* family (Lu et al., 2020). The biological nature of CLasMV1 phage remains unknown. For further phage characterization, it will be beneficial to observe and obtain phage particles. In an attempt to bypass the CLas unculturable barrier, transmission electron microscopy (TEM) was used to examine CLas-infected plant samples, and phage particles were not observed (data not shown). In the SC1 and SC2 phage study, phage particles were observed by TEM (Zhang et al., 2011). However, no procedure for further isolation of CLas phage particles has been reported so far, an indication of technical challenge in CLas phage study. In this study, we demonstrated that mining sequence data via bioinformatics was highly effective and productive for CLas phage research.

While complete integration of CLasMV1 genome sequence in CLas chromosome was not found, some CLas strains contained homologous genes of RIP and MCP of CLasMV1 (Figures 2, 7). It was noted that among these CLas strains, some contained both RIP and MCP genes, and some only contained one of the two genes. In addition, more strains contained RIP genes than MCP genes. All these suggested that the two genes were not integrated in CLas chromosome simultaneously, or if they did, the MCP gene was deleted in a faster rate than that of the RIP gene. It was also reported that the genome of an obligate intracellular pathogen, *Chlamydophila pneumoniae*, contained homologs of RIP and MCP genes of a *Chlamydia*-infecting phages in *Microviridae* (Rosenwald et al., 2014). Mechanisms of phage RIP and MCP gene integrations in bacterial chromosome and their biological functions remain an interesting subject for future research.

Homologs of CLasMV1 RIP and MCP gene were also found in the genome of CLso strains (Figures 3, 4), evidence of horizontal gene transfer (HGT) among species of “*Ca*. Liberibacter” mediated through phages. The relatively high divergency between the RIP and MCP sequences (Figures 3, 4) suggested that the HGT events occurred early, although we could not calibrate the exact timeline. There has been a longtime association between CLasMV1 and its ancestor phage with CLas and CLso. The proposal of subfamily *Libervirinae* was to reflect the current status of the phage evolution and divergency (Figures 3, 4). However, it remains unclear if the RIP and MCP genes in CLso were chromosome-borne or phage-borne or both.

In contrast to the scattered distribution of RIP and MCP genes among CLas strains, a large region of CLasMV1 sequence, equivalent to Contig_63 (1,379 bp), which included ORF2, ORF3, ORF4, and the partial region of ORF1, was found in 26 CLas genomes from different countries (Figure 7). The bacterial genes are usually quickly deleted unless retained for some specific purposes (Moran, 2002). It can be assumed that this sequence contains critical genes to the bacterium despite the unknown functions of the three ORFs. Alternatively, with the conservativeness of Contig_63 among CLas strains (Figure 7), it may have originated from CLas chromosome and was later acquired by CLasMV1. An extensive analysis showed that members of *Microviridae* were able to integrate genes of interest such as peptidase genes from external sources into their genomes (Roux et al., 2012).

Based on the survey of over 1,000 CLas strains collected in southern China, 57% contained CLasMV1 phage, indicating that the phage could likely have an important role in CLas biology. Despite the overall high sequence similarities, it was apparent that strains of CLasMV1 from different sources could vary in the form of SNPs or In/Dels (Figure 6). In addition to HGT, the accumulation of point mutation has been thought to be more important for the evolution of bacteriophage, especially for small ssDNA phages (Székely and Breitbart, 2016), mainly due to their limited genome size and high nucleotide mutation rate (Sanjuan et al., 2010). The diversity of CLasMV1-type phages, revealed by SNPs or In/Dels, indicated that the sequence mutation could be the major force for current evolution of CLasMV1-type phages, although the biological nature of these variations is currently unknown. However, they could set a baseline for future phage diversity study.

## Conclusion

In conclusion, we identified a novel *Microviridae* phage, CLasMV1, with a small circular genome in CLas strain from China. The CLasMV1 encoded eight ORFs, including a phage replication initiation gene, a major capsid gene, and six hypothetical genes. Based on the phylogenetic analysis, CLasMV1 defined a new lineage, designated as subfamily *Libervirinae*, in *Microviridae* family. CLasMV1 was widely distributed with a high copy number in CLas population from southern China. Identification of homologous genes between CLasMV1 phage and CLas strains indicated that the gene transfer could play an important role in early evolution of CLasMV1 phage. The mutation-driven evolution was thought to be the major force diversifying the current CLasMV1-type phage population. The discovery of this novel *Microviridae* phage in CLas strain provided new insights into the biology, diversity, and evolution of *Microviridae* phage.

## Data Availability Statement

Genomes of CLasMV1-type phages has been deposited at DDBJ/ENA/GenBank under the accession number listed in Table 2.

## Author Contributions

LZ, JC, XD, and ZZ conceived and designed the experiments. LZ, ZL, MB, YL, TL, FF, YZ, MX, and ZZ collected the samples and performed the experiments. LZ, ZL, TL, MB, and ZZ contributed to the bioinformatics and statistical analyses. LZ and ZZ prepared the figures and tables and wrote the draft manuscript. JC, XD, and ZZ reviewed and revised the manuscript. All authors contributed to the article and approved the submitted version.

## Conflict of Interest

The authors declare that the research was conducted in the absence of any commercial or financial relationships that could be construed as a potential conflict of interest.

## Publisher’s Note

All claims expressed in this article are solely those of the authors and do not necessarily represent those of their affiliated organizations, or those of the publisher, the editors and the reviewers. Any product that may be evaluated in this article, or claim that may be made by its manufacturer, is not guaranteed or endorsed by the publisher.

## Footnotes

1
http://www.dnastar.com
