# Supplementary material for: A Novel Microviridae Phage (CLasMV1) From “Candidatus Liberibacter asiaticus”
Source: Front Microbiol. 2021 Oct 13;12:754245. doi: 10.3389/fmicb.2021.754245 (PMC8548822; doi:10.3389/fmicb.2021.754245)
Supplement: Supplementary file 1 [file Data_Sheet_1.PDF]

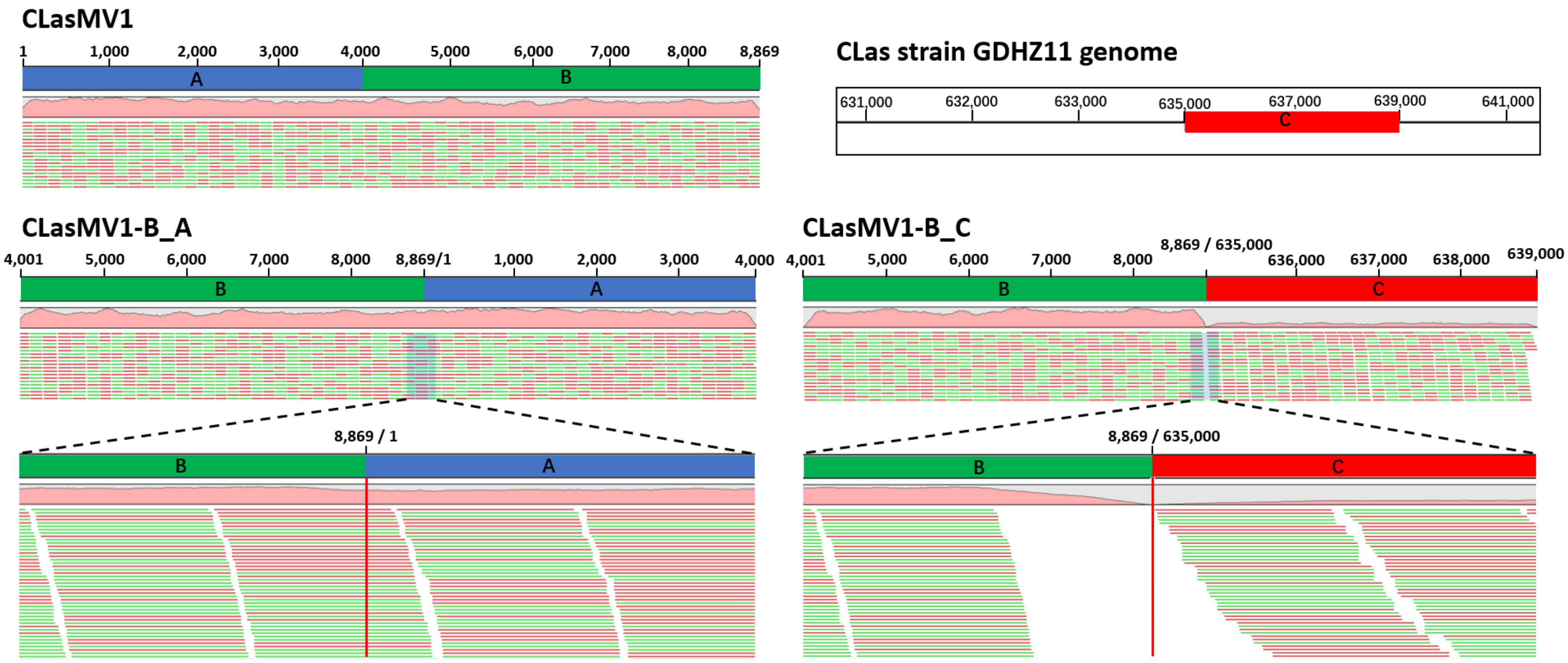

Figure S1. Circularity evaluation of CLasMV1 phage through HiSeq reads mapping. The first 3,000 bp sequence (A region) of CLasMV1 phage was cut and added to the end as a new CLasMV1-B_A sequence. As control, a randomly selected sequence from position 600,001 to 603,000 in "*Candidatus* Liberibacter asiaticus" GDHZ11 genome was cut and added to the end of CLasMV1 as a new sequence CLasMV1-B_C. Both CLasMV1-B_A sequence and CLasMV1-B_C sequence were used as the reference for GDHZ11 Hiseq reads mapping. Note the continuum of reads in CLasMV1-B_A (circular) and the break in CLasMV1-B-C (non-circular).

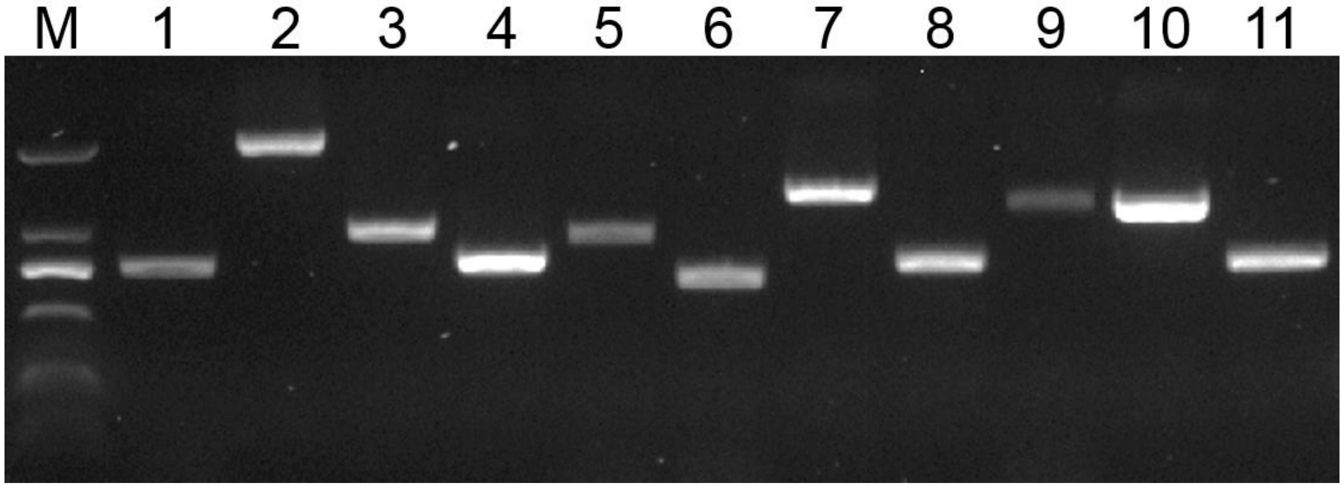

Figure S2. PCR result using primer sets covered the full length of CLasMV1 genome. M, DNA ladder (top to bottom in bp: 2000 bp, 1000 bp, 750 bp, 500 bp and 250 bp). 1, PCLas-1F/PCLas-1R; 2, PCLas-2F/PCLas-2R; 3, PCLas-3F/PCLas-3R; 4, PCLas-4F/PCLas-4R; 5. PCLas-5F/PCLas-5R; 6, PCLas-6F/PCLas-6R; 7, PCLas-7F/PCLas-7R; 8, PCLas-8F/PCLas-8R; 9, PCLas-9F/PCLas-9R; 10. PCLas-10F/PCLas-10R; 11. PCLas-11F/PCLas-11R. The detail information of all primer sets is listed in Table S2.

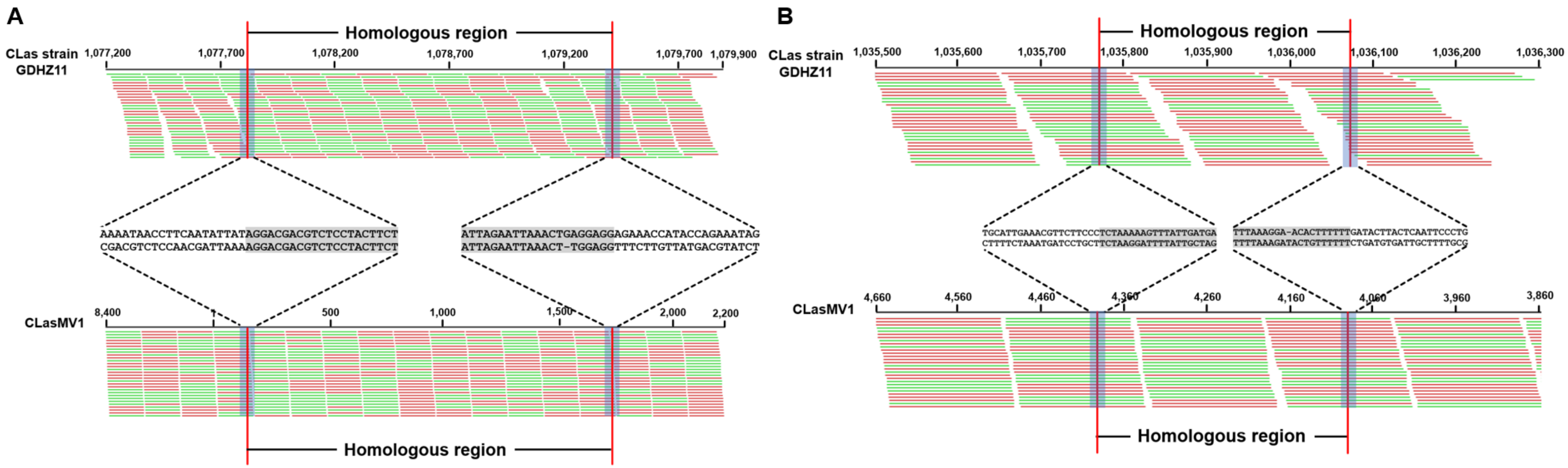

Figure S3. Confirmation of the presence of two homologous regions between "*Candidatus* Liberibacter asiaticus" (CLas) strain GDHZ11 and CLasMV1 sequence through HiSeq reads mapping. A, the homologous region located at the nucleotide position 91 to 1,752 of CLaMV1 genome (CP045566) and the nucleotide position 1,077,726 to 1,079,418 of CLas strain GDHZ11 chromosome (CP045565). B, the homologous region located at the nucleotide position 4,091 to 4,382 of CLasMV1 genome and the nucleotide position 1,035,779 to 1,036,070 of CLas strain GDHZ11. The homologous region is marked between two red lines. The junction region between the homologous region and CLas/CLasMV1 sequence is highlighted by a blue shadow. Noted the unique junction regions are covered with continuous reads, indicated the presence of the homologous region in both CLasMV1 genome and CLas strain GDHZ11 chromosome. Sequences of junction regions are aligned and homologous sequences region is marked with grey.

Table S1. Sequence of bacteria strains and phage isolates used for analysis in this study.

No.	Name of strain	Accession number	Purpose
1	*Escherichia virus* phiX174	NP_040711.1, NP_040703.1	For phylogenetic analyses
2	*Enterobacteria* phage alpha3	NP_039597.1, NP_039590.1	For phylogenetic analyses
3	*Escherichia* phage phiK	NP_043949.1, NP 043942.1	For phylogenetic analyses
4	*Escherichia* phage St-1	YP_002985212.1, YP_002985204.1	For phylogenetic analyses
5	*Escherichia* phage NC35	YP_512406.1, YP_512399.1	For phylogenetic analyses
6	*Escherichia* phage FL68	AGS81871.1, AGS81863.1	For phylogenetic analyses
7	*Escherichia* phage ID18	YP_512796.1, YP_512788.1	For phylogenetic analyses
8	*Escherichia* phage ID11	AAW72844.1, AAW72838.1	For phylogenetic analyses
9	*Spiroplasma* phage SpV4	NP_598320.1, NP_598335.1	For phylogenetic analyses
10	*Bdellovibrio* phage phiMH2K	NP_073538.1 / NP_073537.1	For phylogenetic analyses
11	*Chlamydia* phage Chp1	NP_044312.1, NP_044320.1	For phylogenetic analyses
12	*Chlamydia* phage Chp2	NP_054647.1, NP_054653.1	For phylogenetic analyses
13	*Chlamydia* phage	NP_510872.1, NP_510879.1	For phylogenetic analyses
14	*Chlamydia* virus CPAR39	NP_063895.1, NP_063900.1	For phylogenetic analyses
15	*Microviridae* sp.	AXH74991.1; AXH76180.1; AXF51979.1; AXF51980.1; QJB21683.1; QJB20387.1; QJB20415.1; QJB21619.1; QJB20797.1	For phylogenetic analyses
16	*Sinobacteraceae bacterium*	TXG98908.1; TXG98910.1	For phylogenetic analyses
17	*Pseudoxanthomonas suwonensis*	PZO60394.1	For phylogenetic analyses
18	Tortoise microvirus 88	QCS37339.1	For phylogenetic analyses
19	*Citromicrobium* phage vB_Cib_ssDNA_P1	ATW62973.1; ATW62975.1	For phylogenetic analyses
20	*Novosphingobium tardaugens*	WP_021691789.1	For phylogenetic analyses
21	*Sneathiella* sp.	PHQ69335.1; PHQ69336.1	For phylogenetic analyses
22	"*Candidatus* Liberibacter asiaticus"	WP_045490387.1; WP 015452965.1; OMH86567.1	For phylogenetic analyses
23	*"Ca.* L. solanacearum"	WP_034441566.1; WP_045960446.1; WP_045961120.1; WP_103847521.1; WP_013462096.1; WP_013462103.1; WP_013462099.1; WP_013462108.1; KJZ81438.1; KGB27738.1	For phylogenetic analyses
24	Liberibacter phage SC1	HQ377372.1	For genome assembly and identification of novel contigs
25	Liberibacter phage SC2	HQ377373.1	For genome assembly and identification of novel contigs

26	Liberibacter phage P-JXGC-3	KY661963.1	For genome assembly and identification of novel contigs
27	“*Ca*. L. asiaticus” str. gxpsy	CP004005.1	For identification of novel contigs
28	“*Ca*. L. asiaticus” str. A4	CP010804.2	For genome assembly and identification of novel contigs
29	“*Ca*. L. asiaticus” str. psy62	CP001677.5	For identification of novel contigs
30	“*Ca*. L. asiaticus” str. JXGC	CP019958.1	For identification of novel contigs
31	“*Ca*. L. asiaticus” str. Ishi-1	AP014595.1	For identification of novel contigs
32	“*Ca*. L. asiaticus” str. AHCA1	CP029348.1	For identification of novel contigs
33	“*Ca*. L. asiaticus” str. FL17	JWHA00000000.1	For identification of novel contigs
34	“*Ca*. L. asiaticus” str. YNJS7C	QXDO00000000.1	For identification of novel contigs
35	“*Ca*. L. asiaticus” str. YCPsy	LIIM00000000.1	For identification of novel contigs
36	“*Ca*. L. asiaticus” str. LBR19TX2	VTMA00000000.1	For identification of novel contigs
37	“*Ca*. L. asiaticus” str. LBR23TX5	VTMB00000000.1	For identification of novel contigs
38	“*Ca*. L. asiaticus” str. SGCA16	VTLZ00000000.1	For identification of novel contigs
39	“*Ca*. L. asiaticus” str. DUR1TX1	VTLT00000000.1	For identification of novel contigs
40	“*Ca*. L. asiaticus” str. Mex8	VTLU00000000.1	For identification of novel contigs
41	“*Ca*. L. asiaticus” str. SGCA5	LMTO00000000.1	For identification of novel contigs
42	“*Ca*. L. asiaticus” str. CHUC	VTLV00000000.1	For identification of novel contigs
43	“*Ca*. L. asiaticus” str. GFR3TX3	VTLR00000000.1	For identification of novel contigs
44	“*Ca*. L. asiaticus” str. HHCA16	VTLY00000000.1	For identification of novel contigs
45	“*Ca*. L. asiaticus” str. MFL16	VTLX00000000.1	For identification of novel contigs
46	“*Ca*. L. asiaticus” str. DUR2TX1	VTLS00000000.1	For identification of novel contigs
47	“*Ca*. L. asiaticus” str. CRCFL16	VTLW00000000.1	For identification of novel contigs
48	“*Ca*. L. asiaticus” str. TX1712	QEWL00000000.1	For identification of novel contigs
49	“*Ca*. L. asiaticus” str. SGpsy	QFZJ00000000.1	For identification of novel contigs
50	“*Ca*. L. asiaticus” str. AHCA17	VNFL00000000.1	For identification of novel contigs
51	“*Ca*. L. asiaticus” str. JXGZ-1	VIQL00000000.1	For identification of novel contigs
52	“*Ca*. L. asiaticus” str. GDHZ11	CP045565.1	For identification of novel contigs

Table S2. General information of primer sets used in this study.

No.	Primers	Sequence (5'-3')	From	To	Product size	Amplicon sequence obtained by Sanger Sequencing*	Purpose
1	PCLas-1F/PCLas-1R	CAGTCGGTGCCAAAAGAACA/CTCGTGCGGTGTTCTGATTC	8,563	473	780	CAGTCGGTGCCAAAAGAACAGGCTGCAACTGAAAAGGATAAAAAGCACCTGCAACAGGATAAA<br>CAGGTTTACGACGTTGCTTCGGTGCACTCGCCATCGAACTAATACTCTTACCTTGAGTATGCTCCA<br>AAACCTTTACTTCTTTAGTTTTAGTATCAGCCATAAAAAAATCCTATAAGAGAGAACGTTTAATC<br>ACTCTAAATTGACGAGGAAGGGTAGGAATATTATCAGTAGTAGTCACACGCCGACGAGGAACAT<br>TCCCAGATCGAACATATCTTCTTCTATTCCATCCTCGTCCTCTACCGTAATAAGGACGACGTCTCC<br>AAGTATAAGGACGACGTCTCCAAGCATAAGGACGACGTCTCCAAGTATAAGGACGACGTCTCCA<br>ACGATTAAAAGGACGACGTCTCCTACTTCTTGCGAAATAAGGTCTACGACGTCTGTTATAAGGAA<br>GTCTCTTATAAGGTCTATTAAAACGTATACGATATGGACTAACAATCCTAGAAGACCCATACGCA<br>GGTTGAAGTCGTCTTCGATACATATTCATCCTAGAATAAGTATTAAAAGGTCGGGCTCTTCTTGA<br>TGAAGTGTAAGGACGACGACGCCAACGACGCTGTGTAAAGTTAGGAGAGTAACGATATCGATAA<br>GAAATAGCCATAATAAAAAAACCTTAATAAATAACCTTAATAATTAACCTCAATAAATAACATT<br>AAATAAATTAATGTATTACTCTGTACCCTCCTTAGCTTCCTCAGATTCAGAATCAGAACACCGCA<br>CGAG	Confirmation of junction region between contig63 and contig3
2	PCLas-2F/PCLas-2R	AGTAGTAGTCACACGCCGAC/TCAATAAGGATGATAAGGCGGAA	8,792	1,918	1,996	AGTAGTAGTCACACGCCGACGAGGAACATTCCCAGATCGAACATATCTTCTTCTATTCCATCCTC<br>GTCCTCTACCGTAATAAGGACGACGTCTCCAAGTATAAGGACGACGTCTCCAAGCATAAGGACG<br>ACGTCTCCAAGTATAAGGACGACGTCTCCAACGATTAAAAGGACGACGTCTCCTACTTCTTGCGA<br>AATAAGGTCTACGACGTCTGTTATAAGGAAGTCTCTTATAAGGTCTATTAAAACGTATACGATAT<br>GGACTAACAATCCTAGAAGACCCATACGCAGGTTGAAGTCGTCTTCGATACATATTCATCCTAGA<br>ATAAGTATTAAAAGGTCGGGCTCTTCTTGATGAAGTGTAAGGACGACGACGCCAACGACGCTGT<br>GTAAAGTTAGGAGAGTAACGATATCGATAAGAAATAGCCATAATAAAAAAACCTTAATAAATAA<br>CCTTAATAATTAACCTCAATAAATAACATTAAATAAATTAATGTATTACTCTGTACCCTCCTTAGC<br>TTCCTCAGATTCAGAATCAGAACACCGCACGAGTTCTGACTCTGAATTCCTATGAAAATCTGAAA<br>TCAAAGACCCAAACTTATCCAAATAAGATTTCAGATTATCAACTAACAAATGCTTATTTAATAAC<br>AAATAATCATAACGCTGAGTTAGATCAGAAAGTTCATCAGAAGTAATCTGATCACTATCCGAAG<br>CAAAGTCCAAATCCAAAAGAACAGAATATCTCTCTTCCGCATACGCCTCACACATTATTTCACTC<br>ATTTCTTCTTTTCCTTAGTTACCTCATACCAAACAGCTACTTCATCCGAAACAAACGAATGAAAAC<br>GGGAAACATAATCTAGAGAAAAATTATAAACAGGAAGATCTCTCAAATATTTTAAACGCTCCCA<br>CAAATCATTAACAAAAGAATAATCAGACGAACCATTTTTCTCTAAATCATCCAAAATAGCATCGC<br>GCTCTTCTACAGAAAGCATCGCTAAATCACTCATAGAAGGCTTTGCCTTTCGCAAAGCCTCCACA<br>ATAGAATTACGCTCTAATCTCCAATACTCTATAAACTCACGATTACGAGAAACATCAGTATTCAT<br>AAAAAAATCCTCTCCCAAAAAACCTAAGGACTACCAGGAGCATTCAATTTATTAAGATCAACCTT<br>CAATTCAGAAGAGGAATTTTTTTCATCAACTTTATCCTCCTTAACTAAATGAGAATGAGGATAAA<br>CAACATCGATAACAGAAGGACCAACTTTTTTAGGATATTTAGCAAAGTAAAAACTCATACTCCGT<br>CTATGCGCTTCTAAATCAGAAAGACGAGTTTTCAAATATTCGAAAACTTCAATACTCTCAACATC<br>CTGTAAATCCTGATCCAAAGCATTAATAATATCACCACGCTCAGACCAATAAGTCCTTCTAACAG	Confirmation of junction region between contig63 and contig3

						AACTCGAAAAAACATTACTGTTAGGAATATGAGGACCCACAACTCGCGCAACTTTCTTGTAACGCTCAAGAGCCTCATGCACATCAGCAAAACTTTTACGCTGAACAAAATGGGAAACAAAACAAAATACAAGAATAGAAACCTGTACCAGGTCATTCCAAGTAACAGAAAGATACGTCATAACAAGAAACCTCCAAGTTTAATTCTAATTCTGATATGTTAGTTCAAGCGGATGTTGATCGTGCTTTTTCTGAAATGTATGGCAACTCTGAACAAGCTGCCGAAAAAAATTCCTCTTCTGAATCTAAATCTAAAGCTGATCTTAATAAAATGAATGCTCCTGATAGTTTGCCTTCGTAGTTTTTAGATAGTTATAATTGTCGCATAATAAGTATTATCGAAACTATAGGTAGTAAATAACATAAACTAAAAAAATTAACAAGAGGTTAAAAATCAACAAGCTCAATAATAACAGGGTATCCTTCAGAATCAATCTTAGTATATTTGTAGTTATGCCGTTTTATACTCTCGTATATTCTCTCTTTTTCCGCCTTATCATCCTTATTGA	
3	PCLas-3F/PCLas-3R	TTTCGCAAAGCCTCCACAAT/GCGGGTCATGTAACTAAGCG	942	1,940	999	TTTCGCAAAGCCTCCACAATAGAATTACGCTCTAATCTCCAATACTCTATAAACTCACGATTACGAGAAACATCAGTATTCATAAAAAAATCCTCTCCCAAAAAACCTAAGGACTACCAGGAGCATTCAATTTATTAAGATCAACCTTCAATTCAGAAGAGGAATTTTTTTCATCAACTTTATCCTCCTTAACTAAATGAGAATGAGGATAAACAACATCGATAACAGAAGGACCAACTTTTTTAGGATATTTAGCAAAGTAAAAACTCATACTCCGTCTATGCGCTTCTAAATCAGAAAGACGAGTTTTCAAATATTCGAAAACTTCAATACTCTCAACATCCTGTAAATCCTGATCCAAAGCATTAATAATATCACCACGCTCAGACCAATAAGTCCTTCTAACAGAACTCGAAAAAACATTACTGTTAGGAATATGAGGACCCACAACTCGCGCAACTTTCTTGTAACGCTCAAGAGCCTCATGCACATCAGCAAAACTTTTACGCTGAACAAAATGGGAAACAAAACAAAATACAAGAATAGAAACCTGTACCAGGTCATTCCAAGTAACAGAAAGATACGTCATAACAAGAAACCTCCAAGTTTAATTCTAATTCTGATATGTTAGTTCAAGCGGATGTTGATCGTGCTTTTTCTGAAATGTATGGCAACTCTGAACAAGCTGCCGAAAAAAATTCCTCTTCTGAATCTAAATCTAAAGCTGATCTTAATAAAATGAATGCTCCTGATAGTTTGCCTTCGTAGTTTTTAGATAGTTATAATTGTCGCATAATAAGTATTATCGAAACTATAGGTAGTAAATAACATAAACTAAAAAAATTAACAAGAGGTTAAAAATCAACAAGCTCAATAATAACAGGGTATCCTTCAGAATCAATCTTAGTATATTTGTAGTTATGCCGTTTTATACTCTCGTATATTCTCTCTTTTTCCGCCTTATCATCCTTATTGAAACGCTTAGTTACATGACCCGC	Confirmation of junction region between contig63 and contig3
4	PCLas-4F/PCLas-4R	CGCTTAGTTACATGACCCGC/TGGCAGTACAGACGATGGAA	1,921	2,723	803	CGCTTAGTTACATGACCCGCTATCGTATAACGATCAGGCCTAATAAAGAAATCAGGATGAACGGCGCGCCAAGCCGCATCCCTTTTCTTTTTCTCTCTCGCCAACTTAGCCTGAAACCTCCGATATCTCTCTCGATCAGCAGCAATACCTCTTTCATGAACAGTTAGAGGATGCTCATACATAGGTTTATACGCAGGTTTAGGCGGATTGAAATAATCATTTATATTTTTAACAACACTAGTTCTCTTCAAATAATCAAAAAGGTACTGATTCGAAACTTTAAAATCACCTTTCTTAACGACCACATGACCAGACTTATTCTTAATATCTTCTTTATGACGCTCATACAAATGAGATCGCATATAATCAGTCAACTCCTTATACGCATCAGAATGCTTAATAGTTTCCTTAAAATCCTTCAAAAAACCATTTTCAGAACCAGAAACACGCATATAAAGATCATCAATAGTATTAACCACATGAGCCGTACTCAATGGCCTATCAGAACCAAATTTATCTTCTATACCAAGCTTCTTAGCAACTTCAAACTTCTTATATTGATAAGACAAACTATCTAAACCAAAAAAATGAGGAATTCCAAGCTTCCTAGAAACCTTAGACATAACACGACCAAAAAAACCTATATATCGATCAGTCACTTCATCCCAAAGGGCATTTTCCAAAACCTTCACAAAATCAAGTTTATCACCATGTTTAAACACTTGCCTCCACATAACCATAGGATCAATGGTTTTATTATGCCACTCACCTGTTTTCTTATCCTTCCATCGTCTGTACTGCCA	Phage sequence confirmation
5	PCLas-5F/PCLas-5R	TTCCATCGTCTGTACTGCCA/CTATGGTGTGGAGTCGGTGT	2,704	3,663	960	TTCCATCGTCTGTACTGCCACGGCAAAGCATTATAAAACCGCTTAGTATTTTCATCAATTTCCTGATATCTTCCATAAAAAGCACGATTATGCATTGTAAAAGGATTAAAAAAATTACCAAAACGACTTCT	Phage sequence

						CACTTTGGTCTCCTTCACATGTCTCGGCGGACGAGACAAATTATGAGGAGCAAATTCTGTTGCAA<br>GCACTCGAAAACCTTTAGAATTCAAAACACCATAAATCCTATAAGCAGGTAACTGATATGGCTTT<br>AAACGAGGTAACGGTAACGATTTAGGCTCACCATCAGGTAAATAAGGAAAATAAGGAGAAACA<br>TAAAGAGGTTTCACCTTCGAACCATCAATAGGTTTCTCAGGCGAACCAGCCTGAGACTGCGAAAC<br>AACCGCAGGCGCAGGTTCCGTCTGAATCTCAGGAAAATTACCAAACAAAGGTTTCGTCTGAATCT<br>TAGGTGTAAAAACCGAAGGTATCGTATCAATCTGAGATAACATAAAAACCGAAGGCTTCGTCTG<br>AATCTGAGACTCCACCTTCGGCGGCGCAAAAATCATAGATTCCATTTGCGGAATAAACAAAGGTT<br>TAGGTTCAGCATAACGCCGATTATGTTCATGCACAGCACGATCAAACGTCGCCTGCACTTGGGTC<br>TTTAAATGCGCACCACCATCTAAAATTTTATCAGGATAAGCATTATAAAGATCCTTTAAAGAACG<br>CTTCGCTAAAGCACGAACAGAAGGCTTAACCTTCGGCAACTCAACAGTTTGAGGTAAAGAAGGA<br>TCAAGAGCAGGCCTAACCTTCGCCGGATGCAACGGAGATACAGCCACACTAGACAACAACGGAC<br>GAGGGAAATAAATATCCTCTAATTGCTTAAAAAATTCAACAGGATTACTCTGATACAAAGATTTA<br>GGAGGAAGTTTAGGCAATAAACCACAAAAAATCCACACCGACTCCACACCATAG	confirmation
6	PCLas-6F/PCLas-6R	GCCACACTAGACAACAACGG/ACGTGCTGCTGTTTTGATGT	3,523	4,230	708	GCCACACTAGACAACAACGGACGAGGGAAATAAATATCCTCTAATTGCTTAAAAAATTCAACAG<br>GATTACTCTGATACAAAGATTTAGGAGGAAGTTTAGGCAATAAACCACAAAAAATCCACACCGA<br>CTCCACACCATAGGAGACATAATAATCTCCTAAACCAATAGACTTAAGGAAAAAAGGATAATCC<br>TTTTCATCAATACACTTTCTAAGATAACGTAACTGAGAAGCTATAAAATCAGGAAGTTTACTAAT<br>AGGAGTCCAAACAGCAGGCATAAGAGAATCAGCCCATGATTTCTGATAATGATGATAAAAACCT<br>GCAATACCATAAGTCACCGTATAATAATGAGGATCCAATTCATCATCATAATATAAATACTGACG<br>TGTCACAGACATAGGTCCATGAAAATCATAACCAGAAAAATCAATACGAAGATCACTAAGAGCG<br>TCAGGTAAATCTTTTGGTTCAGGCTCCAGAAGCTTTGCTACACGCTTAGCAGCAGTCGTTGCCTTT<br>TCCAAAAGATCAAAATACTTATTCAAAATCTTCGCAAAAGCAATCACATCAGAAAAAACAGTAT<br>CTTTAAAAGAAACAGAAAAATCCACCAACAAACTATCAAGATTAAAAACACCATCTCGAAGATA<br>CCGACTTAAAACGGCAACTCCTAAACTAGCAAATGGTGCAACCCACATCAAAACAGCAGCACGT	
7	PCLas-7F/PCLas-7R	TAGGAGTCCAAACAGCAGGC/CCTTGACTAGAACCCAGCGA	3,779	5,078	1,300	TAGGAGTCCAAACAGCAGGCATAAGAGAATCAGCCCATGATTTCTGATAATGATGATAAAAACC<br>TGCAATACCATAAGTCACCGTATAATAATGAGGATCCAATTCATCATCATAATATAAATACTGAC<br>GTGTCACAGACATAGGTCCATGAAAATCATAACCAGAAAAATCAATACGAAGATCACTAAGAGC<br>GTCAGGTAAATCTTTTGGTTCAGGCTCCAGAAGCTTTGCTACACGCTTAGCAGCAGTCGTTGCCTT<br>TTCCAAAAGATCAAAATACTTATTCAAAATCTTCGCAAAAGCAATCACATCAGAAAAAACAGTA<br>TCTTTAAAAGAAACAGAAAAATCCACCAACAAACTATCAAGATTAAAAACACCATCTCGAAGAT<br>ACCGACTTAAAACGGCAACTCCTAAACTAGCAAATGGTGCAACCCACATCAAAACAGCAGCACG<br>TTTTAAAAACTTACCTGCAACAATCTCAACAGAATACGCATCCACACACGCAACATACCGAACA<br>AAATTCTTACAATTATCATTACTAGCAAACATAAATCTATAAGCTGGAGACACTTTAGATTTAGT<br>CAAACTAGCAATAAAATCCTTAGAAGCAGGATCATTTAGAAAAGATCTAAAATGAGCAGGTTTA<br>GCCTTAAGTTTACCACGAAATCTATCCATAAAAACAACCAAAAGTAATAATAAATTAAGAATAC<br>CACTGTATCATTAAAATACAGTAGCGAGGGGAAAATGCATACAAATAAAACAATACTCATAATA<br>ACAGCAGCATTACTTTACTCATGTAGTAGTGAAAACCTATCAGGACAAAACTGCGAATTCCAAA<br>AACTATACAACGATGAATTTCCTTTATTCTTAGAAAAAAAATTCAAAATAAGAAACGTAATGACA<br>GATCTCACTAAAGATCAAGAAAAAATGATATTCACTCTCGAAAAAAAAGTAACCGAAAATATAA<br>AACATGAAATGGGTGAAGATAAATTCATAGAAATGAATCACAAATATGACTTCTTAAGAAAACG	Phage sequence confirmation

						TTTCAACGATTACAACGATATAACAGAAGTATACAACCATCTAAAAAATCCATAAAGTTCACTGC<br>CGACCCTCTGACTACCCTCGCTTCCAATGCTTTCCAGCCTCGGGGTGCACCGTCATCGGGATTAG<br>GCGCTTGTCGCATCTTCGATGCTCCTTCTTCGCGCCTGAGCGCTCGCTCCGTGGGGTTCTCGGCCC<br>TTCGTGGGGTTGGGGGCGCTGCGCGGGCTCGCGCTGCACGGGCCTTTCCTCTGCTTCGCTGGGTT<br>CTAGTCAAGG	
8	PCLas-8F/PCLas-8R	CTGACTACCCTCGCTTCCAA/ACTTTAACCATGACGCCTGC	4,880	5,654	775	CTGACTACCCTCGCTTCCAATGCTTTCCAGCCTCGGGGTGCACCGTCATCGGGATTAGGCGCTTGT<br>CGCATCTTCGATGCTCCTTCTTCGCGCCTGAGCGCTCGCTCCGTGGGGTTCTCGGCCCTTCGTGGG<br>GTTGGGGGCGCTGCGCGGGCTCGCGCTGCACGGGCCTTTCCTCTGCTTCGCTGGGTTCTAGTCAA<br>GGAGCTTCGCCAATTTTCATTTTCTCTAGGAATATTTCATATTCCACTCTGGCCTTTCGGCCAAGC<br>CTCGGTTACCCCTCGGATGGTGTTAAAGCACCATATTGAAAGGACGCTCTTACTCTAATGCCTTTC<br>ATACCTTCTTTCATCAGGTATTTACATACGTAACGGGCAGTGAACAAATCTTCTTTCAGTAATCGA<br>ACGTGAGAAAATCCTTCTCTACTCCATTCCTCTTGAACTTCAGCCTTTTTAAGGAGATTATCACAT<br>TGTTGATGGATCAACATATGAACGTGGGGATCACCACTCTTATGTTTTTCAAATACAAAAAAATA<br>TCTAAATTTCTTACCAGTATTCTTTCGAAGTCGTTTCAAAAATAATGAAACCTTATTACCAAATCC<br>TTTGCATAAAAGAGAAAAAAGAACATTTTCACCAAAAATATCCTTCTTACGCATCAAATGAATCT<br>CAGTAGGACGACCATCTACATTTAGCAAATCACGTTCATGAGGAGGAAAAGAATCAATATAATT<br>AAAAACCATCGAACGATGATTAGCAAAATGATTAGCAGGCGTCATGGTTAAAGT	Phage sequence confirmation
9	PCLas-9F/PCLas-9R	AATGAATCTCAGTAGGACGACC/AGTGAATGAGTTTGCTGCGA	5,528	6,735	1,208	AATGAATCTCAGTAGGACGACCATCTACATTTAGCAAATCACGTTCATGAGGAGGAAAAGAATC<br>AATATAATTAAAAACCATCGAACGATGATTAGCAAAATGATTAGCAGGCGTCATGGTTAAAGTT<br>ACAAACCATGTACGCGTAGAACGTTTCACTTCAATATAGGCACGACGTAACCAAAATAAACCTC<br>GGTTCTTATAACAAGAAGAACAACTCCGACAAGGAAGTATCAAATACATAAAACGATGGAAATA<br>AGAATAATGATTAAAAAGTTCAAGCCTATCGGTATCATACAAAACAGAAACAACAGGAAAAAG<br>ACAACGCCTAGAAAAATCTAAACAAAAACAATTATTATTAGGACTATGTTTATAAAATGAAGAA<br>CATCTAAATTGCCAATAAAGTTTATCAAAAACATCCTTCTCTATTAAATTACAATTACCTTTAGGT<br>GAAGTAGAATAAATATCTTTAAAGAGTTTATCATTAGCAGATAAAAAACGATAGCGATGATAAA<br>AATGTTTAGGAAGAATATTACCAGCATAATACTTGAAATAAATATCCATAGGTTCAAAAATGGA<br>AAATAATGCAGGGACTTCATCCTTAGCAAAATACTCACCACCTTTAAAACAATATCGAGAATAAT<br>AAATACAATCTAAAAGATCACAATAATAGGAATAATCATCCGAATATTGAGAACGAGATAAATC<br>AATAGAAAAAATCTTAGCATAAAATTCTATCGACTGACAAAAAGTATCATATAAATCAATACAA<br>TAATTCTTATCATTATGACCATAATTCCACAATTTCATTAAATTTGTATAACGATCATCTAAGTCC<br>ATGAAAGTACACCATAAATAAAAAAGGGAAAAAAAGGTGTCACTTGTGCTTATATATATCAAGT<br>ACTCGCTTCGCGAATATATACGCACAATACTAATTCCCAACTGTAACTGTAGCCTGTGGAAAGCC<br>TACAGTTGGGGAGGCTTGCGCCTTCCCACAAGGCTACAGTGTTACTTTTTCTTAGGGATAGGAAA<br>AAGAGGAAGACTTAAACTTCTACCAAACCCATTACCAATTCTCATTAATCTCTGTTCAAGAGTAT<br>CAAGCTCAAACAAAGCATCACGCTTCTCAAGTTCACGTTTTTCAATATCACGAATCAAACTCTGT<br>AACCGATTCCGTTTTTTACGATCACGAGTCGCAGCAAACTCATTCACT	Phage sequence confirmation
10	PCLas-10F/PCLas-10R	CACGCTTCTCAAGTTCACGT/GGTGTGATGATCATGCGACC	6,641	7,834	1,194	CACGCTTCTCAAGTTCACGTTTTTCAATATCACGAATCAAACTCTGTAACCGATTCCGTTTTTTAC<br>GATCACGAGTCGCAGCAAACTCATTCACTAACTTCTTTCTCTCATTCTCAAGCTTATAAATATCCT<br>GTTCGAATTCATCCTTAAGCTTATAAATATCCTGTTGGAGTTCATCATCAGACTTAGGTTTAGGTT<br>TCTCAACCACTTTAGGTTTCTCAACCACTTTAGGTTTCTCTACCCTAGGTTGAGGCCTCTCCATTTT	Phage sequence confirmation

						CTGAGGAGGTGGTGGTGCAGGAGGAACCACAGGTTCAACTTTCTTTTCGACAGGCTGAGGCTTCTCCTCCACTTCAGGAATTATCAGTGGAGGAGGTGGAGGAACCACCACAGGTGCTGCAGGTGGAAGAGAAGGCGGAGGTAAAACCGATATAGTTTCGGATACAAGTGGAGGTCGTACTTCTACTCCTTGCTCAATCTTATCCTCCGCCTTCTTTTCACCCTGAACTTCTTCCAGTACTGGTTTATCTCTCGATAATAAATCAGAAGAATCTGCAGGGGGTAAAGACACAGTGCTAACCACAGGGGGCACAGCTGTAATAGAATCACTGACGCGAGTAGGAACAATACCATCGGTAATAAATTGTTGTTTCTCATCCTTGACCTCCCAATCTAATTTTTTTTTGAGTCTTGAATCGTACGAGTAATAGGAGTTGTACCAACTTGAGACTGTAACTCTGACTTCGGCTTACACTCACCAGTAGTAGGATCAAAGTCATGATACTTATATTCAATATCTTGACGATTATCTTCATTAACTCTAGGAACACGGGATTTAACGGCCATCCTCACAATACCTTCATATTTAAAAGGATGTGAAACACCATCATATAAAGAACCAATACCAGCATCAGTAGGATAACAATCCTTACCAAAATTAATAGAATTCTTCACAACAGTATACGGATCCTTCTCCGAAGGAACACTTATAAACTGCTCACCTCGACGATATAAATCAGCAAAATTAATATCATAAGAATCATCTCTACTGTTCGCATAAGGACCTTGGTCCTTATTATATTTGACAACACCATGACGATAGGCAGAATCCAATAACTCAGGAAGAAACCAATTCATATTCGTCATATAAGAAGTATGACTACTAATCTGATCCTTAGCAAAAACCTTCGGTCGCATGATCATCACACC	
11	PCLas-11F/PCLas-11R	CCTTCGGTCGCATGATCATC/TGTTCTTTTGGCACCGACTG	7,810	8,582	773	CCTTCGGTCGCATGATCATCACACCAAGGAAAAATCCAGGATGATTAAGAAACTTGGTCTGACGACCACGACATAATACATGCTTAAAATTAAATTTAAAATTATTAACGTAATCACCTTTCGAAAGATCGAAATGATTACGAGGCATCTCAAAAGAACGATACAATCCTAACAACTCAGGAGGATACTCCTCACGAAGTTTCTCTTCCTCCTTCCTGATTAACTCAACAGTAAGACCAAAATGACGCATATACTGTTTAAAAGTAAATTCAGTCTTTCCAGAAGCATACATACGCTCCCATTCTTTATACTGAGGGCTTTCAGTAGAAACACGTTCAGCCTCTACTGCTTCCTCATCCGTCTGAAAGTCCTGGATATAACCAGAACTATGTATATCACAAATAGGAAGACCTTTATACATATACGCTGAAGCAGCAACACTCTTACCTTCCTTATCTTTAGTCCGATAAAAGAAACTAGTAACAGTCTTCAAACAACGCTCAATAAAATTAATACCACCAGAATGATAATAATTCTTACTTGAAAAAGAAGGATTATCATCTCTCGCTAATTTATCAACGCCAGCAAAATTACCTTGATCATCATACTCCGCCATATTCTTATAGAAATCAGTAATACTAATCAAAGGAACATAAAAAACAGCAAACTCATGCCAATAACCAGCAGAACTCTTCAAGGTAGAAGAAGTAATATTCAAATTAATATCAAAATGCTCAATCGTCTCTTTACCAACAGTCGGTGCCAAAAGAACA	Phage sequence confirmation
12	CLasMV1-1F/CLasMV1-1R	ACGACCACATGACCAGACTT/TGATGCGTATAAGGAGTTGACTG	2,218	2,310	93	NA	CLasMV1-type specific Real-time PCR
12	CLasMV1-2F/CLasMV1-2R	TGCAAGCACTCGAAAACCTT/CCGTTACCTCGTTTAAAGCCA	2,895	2,978	84	NA	CLasMV1-type specific Real-time PCR

*NA, not applicable.

Table S3. Blast result of CLasMV1 against with CLas whole genomes and three phage sequences (SC1, SC2 and P-JXGC-3). The covered of contig = (The total hit length / Contig length) * 100%.

No.	Name	Length	Coverage	Total alignment length	% covered of contig	Similarity of match sequence	Copy number / Regions
1	Contig_8	367,497	68	367,442	100%	100.0%	Single copy in CLas chromosomal region
2	Contig_4	131,513	69	131,514	100%	100.0%	Single copy in CLas chromosomal region
3	Contig_10	110,934	67	110,939	100%	99.9%	Single copy in CLas chromosomal region
4	Contig_54	68,284	67	68,284	100%	100.0%	Single copy in CLas chromosomal region
5	Contig_18	62,308	62	62,317	100%	99.9%	Single copy in CLas chromosomal region
6	Contig_75	48,213	64	48,213	100%	100.0%	Single copy in CLas chromosomal region
7	Contig_16	44,479	61	44,479	100%	100.0%	Single copy in CLas chromosomal region
8	Contig_80	43,252	63	43,252	100%	100.0%	Single copy in CLas chromosomal region
9	Contig_29	55,823	61	55,805	100%	100.0%	Single copy in CLas chromosomal region
10	Contig_69	41,185	65	41,185	100%	100.0%	Single copy in CLas chromosomal region
11	Contig_33	37,948	66	37,948	100%	100.0%	Single copy in CLas chromosomal region
12	Contig_28	31,196	61	31,209	100%	99.6%	Single copy in CLas chromosomal region
13	Contig_1	23,246	65	23,251	100%	99.9%	Single copy in CLas chromosomal region
14	Contig_71	22,437	63	22,437	100%	100.0%	Single copy in CLas chromosomal region
15	Contig_17	20,218	66	20,218	100%	100.0%	Single copy in CLas chromosomal region
16	Contig_86	17,973	69	17,975	100%	99.9%	Single copy in CLas chromosomal region
17	Contig_49	24,656	70	25,005	100%	100.0%	Single copy in CLas chromosomal region
18	Contig_118	10,853	58	10,858	100%	99.8%	Single copy in CLas chromosomal region
19	Contig_26	9,913	61	9,913	100%	100.0%	Single copy in CLas chromosomal region
20	Contig_38	6,148	68	6,146	100%	99.9%	Single copy in CLas chromosomal region
21	Contig_267	5,282	67	5,282	100%	99.9%	Single copy in CLas chromosomal region
22	Contig_56	1,863	65	1,863	100%	100.0%	Single copy in CLas chromosomal region

23	Contig_370	1,789	63	1,790	100%	99.9%	Single copy in CLas chromosomal region
24	Contig_5	5,766	216	5,766	100%	100.0%	Three copies of *rrn* in CLas chromosomal region
25	Contig_212	1,282	127	1,282	100%	100.0%	Two copies of elongation factor Tu genes in CLas chromosomal region
26	Contig_204	1,276	130	1,276	100%	100.0%	Two copies of integrated region between CLas chromosomal region and prophage region
27	Contig_170	2,111	84	2,365	100%	98.7%	Hypervariable prophage genes contained multiple tandem repeat unit in prophage region
28	Contig_63	1,379	342	1,379	100%	100.0%	Both CLas chromosomal region and CLasMV1 phage
29	Contig_3	7,324	305	4,352	59%	99.0%	CLasMV1 phage
